# Unique metabolic activation of adipose tissue macrophages in obesity promotes inflammatory responses

**DOI:** 10.1007/s00125-017-4526-6

**Published:** 2018-01-14

**Authors:** Lily Boutens, Guido J. Hooiveld, Sourabh Dhingra, Robert A. Cramer, Mihai G. Netea, Rinke Stienstra

**Affiliations:** 1 Nutrition, Metabolism and Genomics Group, Division of Human Nutrition, Wageningen University, Wageningen, the Netherlands; 2 Department of Internal Medicine, Radboud University Nijmegen Medical Centre, Geert Grooteplein Zuid 10, 6525 GA Nijmegen, the Netherlands; 3 Department of Microbiology and Immunology, Geisel School of Medicine at Dartmouth, Hanover, NH USA

**Keywords:** Adipose tissue, Glycolysis obesity, Immunometabolism, Inflammation, Macrophages, Oxidative phosphorylation

## Abstract

**Aims/hypothesis:**

Recent studies have identified intracellular metabolism as a fundamental determinant of macrophage function. In obesity, proinflammatory macrophages accumulate in adipose tissue and trigger chronic low-grade inflammation, that promotes the development of systemic insulin resistance, yet changes in their intracellular energy metabolism are currently unknown. We therefore set out to study metabolic signatures of adipose tissue macrophages (ATMs) in lean and obese conditions.

**Methods:**

F4/80-positive ATMs were isolated from obese vs lean mice. High-fat feeding of wild-type mice and myeloid-specific *Hif1α*^−/−^ mice was used to examine the role of hypoxia-inducible factor-1α (HIF-1α) in ATMs part of obese adipose tissue. In vitro, bone marrow-derived macrophages were co-cultured with adipose tissue explants to examine adipose tissue-induced changes in macrophage phenotypes. Transcriptome analysis, real-time flux measurements, ELISA and several other approaches were used to determine the metabolic signatures and inflammatory status of macrophages. In addition, various metabolic routes were inhibited to determine their relevance for cytokine production.

**Results:**

Transcriptome analysis and extracellular flux measurements of mouse ATMs revealed unique metabolic rewiring in obesity characterised by both increased glycolysis and oxidative phosphorylation. Similar metabolic activation of CD14^+^ cells in obese individuals was associated with diabetes outcome. These changes were not observed in peritoneal macrophages from obese vs lean mice and did not resemble metabolic rewiring in M1-primed macrophages. Instead, metabolic activation of macrophages was dose-dependently induced by a set of adipose tissue-derived factors that could not be reduced to leptin or lactate. Using metabolic inhibitors, we identified various metabolic routes, including fatty acid oxidation, glycolysis and glutaminolysis, that contributed to cytokine release by ATMs in lean adipose tissue. Glycolysis appeared to be the main contributor to the proinflammatory trait of macrophages in obese adipose tissue. HIF-1α, a key regulator of glycolysis, nonetheless appeared to play no critical role in proinflammatory activation of ATMs during early stages of obesity.

**Conclusions/interpretation:**

Our results reveal unique metabolic activation of ATMs in obesity that promotes inflammatory cytokine release. Further understanding of metabolic programming in ATMs will most likely lead to novel therapeutic targets to curtail inflammatory responses in obesity.

**Data availability:**

Microarray data of ATMs isolated from obese or lean mice have been submitted to the Gene Expression Omnibus (accession no. GSE84000).

**Electronic supplementary material:**

The online version of this article (10.1007/s00125-017-4526-6) contains peer-reviewed but unedited supplementary material, which is available to authorised users.


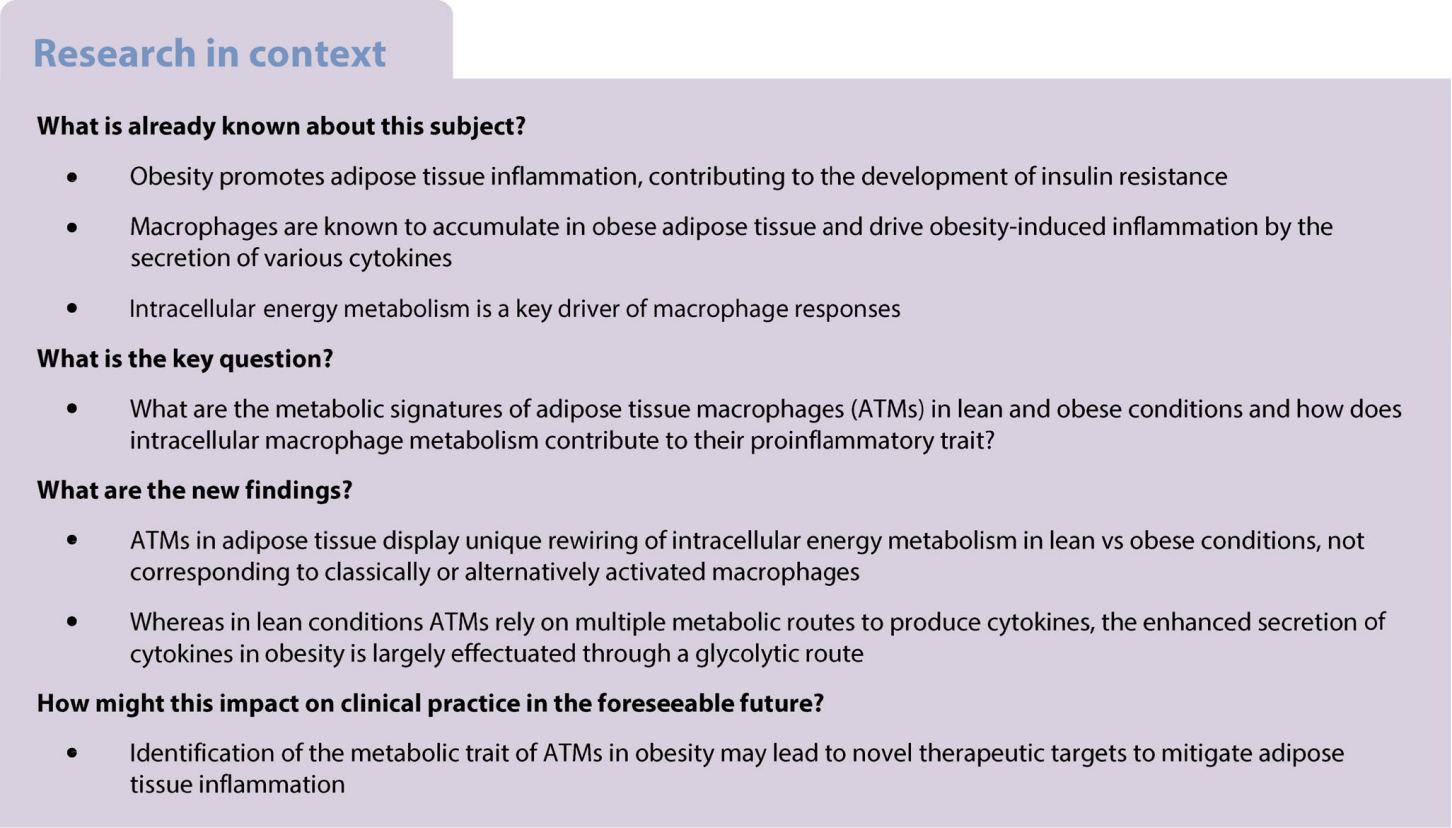


## Introduction

In obesity, macrophages accumulate in adipose tissue and trigger chronic low-grade inflammation which promotes the development of systemic insulin resistance [1–3]. Based on transcriptional profiles and expression markers derived from in vitro experiments, macrophages are generally classified as classically/inflammatory (M1) or alternatively/anti-inflammatory (M2) activated [4, 5]. Applying this phenotypical classification to adipose tissue macrophages (ATMs) has led to the identification of M2 macrophages in lean adipose tissue vs M1 macrophages in obese adipose tissue [6]. In recent years, however, the two-dimensional M1/M2 spectrum has been challenged and macrophages in different tissue environments have been shown to adopt a variety of inflammatory phenotypes that fall outside this classification [7, 8]. Indeed, macrophages in obese adipose tissue display surface-proteins that resemble neither classical nor alternative activation, but rather represent a state of metabolic activation [9]. Intracellularly, ATMs in obese adipose tissue are characterised by lysosomal activity [10], suggestive of robust changes in intracellular energy metabolism of ATM in obesity.

Recent developments in the field of immunology have identified macrophage intracellular energy metabolism as a fundamental determinant of its functional response. M1 macrophages are characterised by a high glycolytic rate whereas M2 macrophages rely mainly on oxidative phosphorylation (OXPHOS) [11, 12]. A central role in driving macrophage polarisation has been appointed to hypoxia-inducible factor-1α (HIF-1α), a master regulator of glycolysis that is critically involved in the development of the M1 phenotype [13, 14]. Profiling of intracellular metabolism in ATMs, as well as identifying key regulators involved, is expected to further the understanding of their metabolic functions and may ultimately bring forward targets for modulating their inflammatory traits. Using various approaches, we identified unique metabolic activation of ATMs in obesity that does not resemble M1 or M2 macrophages. Metabolic activation of macrophages, characterised by increased OXPHOS and glycolysis, was dose-dependently induced during a co-culture with adipose tissue and translated into increased cytokine secretion. Although various metabolic pathways contributed to cytokine release by ATMs, glycolysis accounted mostly for the higher cytokine production by ATMs from obese mice. Inflammatory activation of ATMs during early stages of obesity, however, appeared to be independent of HIF-1α. Further understanding of the functional consequences of metabolic programming in macrophages in lean adipose tissue and metabolic activation in ATMs residing in obese adipose tissue is expected to lead to novel therapeutic targets to curtail inflammatory responses that will ultimately reduce obesity-induced metabolic complications.

## Methods

### Mice

Male C57Bl/6 mice (Harlan, Horst, Germany) were on a high-fat diet (HFD) containing either 45% (D12451) or 60% energy derived from fat (D12492), or on a low-fat diet (LFD) containing 10% energy derived from fat and matching most other components present in either the 45% HFD (D12450B) or 60% HFD (D12450J), for 16 weeks. Mice were stratified based upon body weight at the start of the LFD or HFD intervention. All procedures were approved by the ethics committee for animal experiments at Wageningen University.

For studying the role of HIF-1α in ATMs during the development of obesity, 9- to 12-week-old male C57/Bl6 mice with floxed *Hif-1α* (also known as *Hif1a*) (exon 2) crossed into a background of lysozyme M-driven cre recombinase (LysM *Hif-1α*^−/−^) or C57/Bl6 controls not carrying lysozyme M-driven cre recombinase (LysM *Hif-1α*^+/+^) [15] were exposed to an HFD (D12492) for 8 weeks. After 7 weeks, an insulin tolerance test was performed in mice fasted for 5 h, by injecting insulin (1 U/kg body weight) intraperitoneally. Blood was taken from the tail at specific time points and glucose was measured using Accu-check glucose meters (Roche Diagnostics, Almere, the Netherlands). The study was carried out in accordance with recommendations in the Guide for the Care and Use of Laboratory Animals of the National Research Council. The protocol was approved by the Dartmouth IACUC.

All mice were individually housed and had ad libitum access to food and water. All diets were obtained from Research Diets (New Brunswick, NJ, USA). Experimenters were not blinded to group assignment and outcome assessment.

### Cell culture

ATMs and peritoneal macrophages were isolated from male, wildtype C57Bl/6 mice (Harlan). For details of tissue and cell collection, see ESM Methods.

#### ATMs

Freshly isolated ATMs were cultured in RPMI 1640 (Lonza, Verviers, Belgium) supplemented with 10% (vol./vol.) FCS and 1% (vol./vol.) penicillin/streptomycin (PS) (RPMI/FCS/PS) for 24 h (200,000 cells/well). The contribution of various metabolic pathways to cytokine release was examined by providing 5.5 mmol/l 2-deoxy glucose (2-DG) (Merck, Darmstadt, Germany), 50 μmol/l Etomoxir (Merck), 10 μmol/l UK5099 (Merck) or 10 μmol/l BPTES (Merck) 2 h after plating until the end of the 24 h culture period.

#### Bone marrow-derived macrophages

Bone marrow cells were cultured in DMEM (Lonza) supplemented with 10% (vol./vol.) FCS and 1% (vol./vol.) PS (DMEM/FCS/PS) and 5% (vol./vol.) L929-conditioned medium (L929). After 3–4 days, adherent bone marrow-derived macrophages (BMDMs) were re-plated and exposed for 3 days to an insert containing 25 mg or 100 mg of minced epididymal adipose tissue collected from LFD-fed or HFD-fed mice. Control BMDMs were held in DMEM/FCS/PS containing 5% (vol./vol.) L929 with an empty insert for the same length of time. M1 and M2 macrophages were generated by 24 h incubation with 10 ng/ml lipopolysaccharide (LPS) (M1) or 25 ng/ml IL-4 (M2). For measuring cytokine and lactate production of BMDMs after adipose tissue priming, inserts were removed and BMDMs were held in fresh DMEM/FCS/PS for an additional 24 h. For extracellular flux analysis, BMDMs were scraped, counted and re-plated in a 96-well Seahorse microplate (Seahorse Bioscience, Santa Clara, CA, USA).

#### Adipose tissue

Epididymal adipose tissue was brought into culture and exposed to 17.5 nmol/ml insulin in DMEM/PS for 20 min to measure insulin sensitivity. The tissue was kept in DMEM/FCS/PS with or without LPS (10 ng/ml) for 24 h to measure IL-6 release or was cultured in DMEM/FCS/PS for 3 days for leptin and lactate measurements.

### Extracellular flux analysis

The real-time oxygen consumption rate (OCR) and extracellular acidification rate (ECAR) of ATMs and BMDMs were analysed using an XF-96 Extracellular Flux Analyzer (Seahorse). See ESM Methods for further details.

### Cytokine and lactate measurements

Levels of IL-6, chemokine (C-X-C motif) ligand-1 (KC), TNF-α, IL-1β, IL-10 and leptin in cell culture supernatant fractions were measured with murine DuoSet ELISA Development kits (R&D Systems, Abingdon, UK). An enzymatic assay adapted from the Lactate Assay kit (Merck) was used to determine lactate levels.

### Immunohistochemistry

Paraffin-embedded sections of epididymal adipose tissue were stained with an F4/80 antibody (Bio-Rad, Veenendaal, the Netherlands) and counterstained with haematoxylin. Macrophages were visualised with 3,3-diaminobenzidene (Merck).

### Western blot

Primary antibodies for actin (Merck), AMP-activated protein kinase (AMPK) (no. 2532L), phospho-AMPK Thr172 (no. 2531) and p-Akt Ser473 (no. 4060) (Cell Signalling, Leiden, the Netherlands) were all used at a ratio of 1:1000 according to manufacturers’ instructions, and incubated overnight at 4°C. See ESM Methods for further details.

### RNA isolation and qRT-PCR

RNA from ATMs and peritoneal macrophages isolated from HFD-fed or LFD-fed mice, or from BMDMs, was used for quantitative reverse-transcription PCR analysis (qRT-PCR). The following genes were measured: *Cd11c* (also known as *Itgax*), *Cd206* (also known as *Mrc1*), *Cd36*, *Cd68*, *Glut1*, *Hk2*, *Hif-1α*, *Ldhα* (also known as *Ldha*), *Lipa*, *Pdk4*, *Plin2* and *Vegfα* (also known as *Vegfa*), normalised against *36b4* (also known as *Rplp0*) (ESM Table 1). For further details, see ESM Methods.

### Microarray analysis and interpretation

Four pools of ATMs isolated from epididymal adipose tissue of male C57Bl/6 mice fed an LFD or HFD in four separate experiments were subjected to expression profiling by microarray. In addition, raw transcriptome data from various tissue macrophages, including ATMs (GEO accession no. GSE56682), from LPS-stimulated BMDMs (GSE53986) and from obese diabetic and obese non-diabetic humans (GSE54350) were obtained from the Gene Expression Omnibus. Details of the microarray analysis and interpretation are in the ESM Methods. Microarray data have been submitted to the Gene Expression Omnibus (accession number GSE84000).

### Statistical analysis

Results are shown as mean ± SEM. Statistically significant differences between two groups were calculated using Student’s *t* test. For comparisons between more than two groups, a one-way ANOVA and post hoc Bonferroni’s multiple comparison test was done. When comparing diet and treatment effects within one experiment, data were analysed with a two-way ANOVA with post hoc Bonferroni test (treatment vs control). A *p* value ≤0.05 was considered significant.

## Results

### Unique metabolic and inflammatory activation of ATMs in obesity

**Fig. 1:**
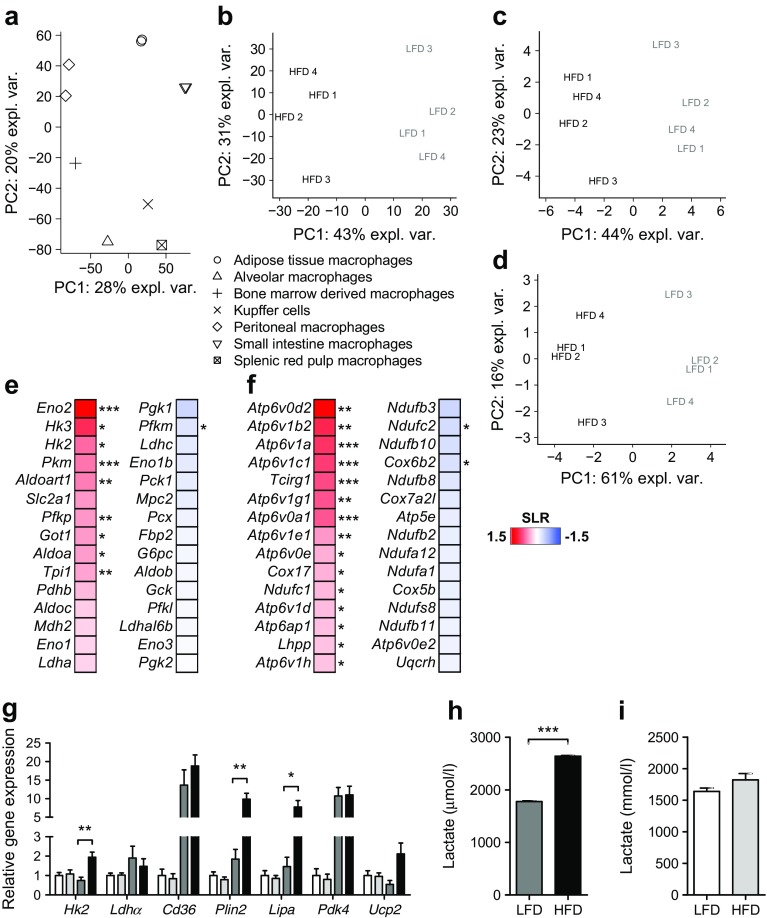
Unique metabolic and inflammatory activation in ATMs during obesity. (**a**) PCA of various tissue macrophages, separated based on their transcriptome. *n* = 2 for macrophages from peritoneum, small intestine and adipose tissue; *n* = 1 for other macrophages. Data are derived from GEO accession no. GSE53986. Percentage of explained variance (expl. var.) is shown. (**b**–**d**) PCA plot of ATMs from obese (HFD-fed) vs lean (LFD-fed) mice (four pools of 4–7 HFD- or LFD-fed mice), separated based on the complete transcriptome (**b**) or on expression levels of inflammatory genes (**c**) or metabolic genes (**d**). (**e**, **f**) Heat maps reflecting expression levels of the top 15 most differentially higher (red) or lower (blue) expressed genes involved in glycolysis (**e**) or OXPHOS (**f**) in ATMs from obese vs lean mice, presented as log_2_ ratio difference (SLR). (**g**) Expression levels of metabolic genes in peritoneal macrophages and ATMs. The ${2}^{-\Delta \Delta {\mathrm{C}}_{\mathrm{t}}}$method was used to determine fold change inductions normalised to *36b4*. Diet-induced changes in expression levels (HFD vs LFD) were tested for significance. (**h**, **i**) Lactate production over 24 h by ATMs (**h**) or peritoneal macrophages (**i**) from lean (LFD) or obese (HFD) mice. Peritoneal macrophages: *n* = 7 (LFD, white bars) vs *n* = 6 (HFD, light grey bars) were used for qRT-PCRs and lactate measurements (**g**, **i**). ATMs: four pools (LFD, dark grey bars) vs six pools (HFD, black bars) of 4–7 mice were used for qRT-PCR (**g**) and a pool of 9 LFD-fed mice vs a pool of 5 HFD-fed mice was used for lactate measurements (*n* = 3) (**h**). Data are presented as means ± SEM. *p < 0.05, **p < 0.01 and ***p < 0.001, as shown by brackets, or for obese vs lean mice in (**e**, **f**)

To examine whether macrophages residing in adipose tissue are transcriptionally distinct from other tissue macrophages, we performed a principal component analysis (PCA) using publicly available gene expression profiles of macrophages isolated from the peritoneal cavity, liver, spleen, lung, intestine and adipose tissue [16]. Indeed, ATMs exhibited unique transcriptomes (Fig. 1a). The presence of obesity clearly affected complete transcriptomes of ATMs further as demonstrated by distinct clustering of ATMs sorted from obese vs lean mice by the pan macrophage-membrane marker Emr1-F4/80 [17, 18] (Fig. 1b). Traditionally, macrophages in obese adipose tissue are characterised by an enhanced inflammatory state [2]. Using inflammatory genes (ESM Table 2) as input for PCA confirmed distinctive inflammatory activation of macrophages residing in obese adipose tissue (Fig. 1c). Interestingly, expression data for genes involved in glycolysis, OXPHOS and amino acid metabolism (ESM Table 2) were also sufficient to distinguish ATMs of obese mice from ATMs of lean mice (Fig. 1d), suggestive of robust changes in energy metabolism of ATMs in obesity. In fact, many genes involved in glycolysis and OXPHOS were upregulated in ATMs from obese vs lean mice (Fig. 1e, f). Noticeably, strong metabolic rewiring upon obesity was specific for ATMs, as these changes were not observed in peritoneal macrophages (Fig. 1g, i). Indeed, metabolic genes were expressed at much higher levels in ATMs than in peritoneal macrophages and were upregulated in obesity (Fig. 1g). In support of our gene expression data, ATMs derived from obese vs lean animals produced more lactate ex vivo (Fig. 1h), reflective of higher glycolytic rates. By contrast, no robust difference in lactate secretion was found in peritoneal macrophages isolated from obese vs lean mice (Fig. 1i).

### Metabolic and inflammatory activation of macrophages in obese adipose tissue is distinct from classical activation by LPS and associates with the presence of type 2 diabetes in obese humans

**Fig. 2:**
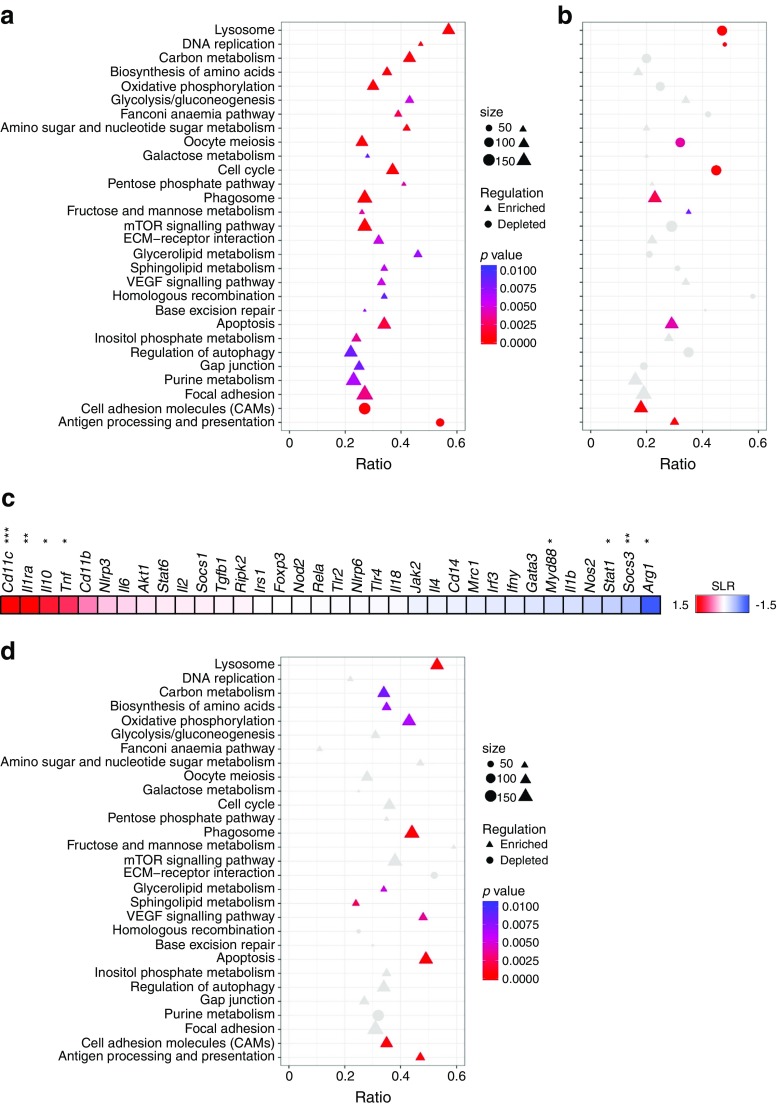
Metabolic and inflammatory activation of macrophages in obese adipose tissue is distinct from classical activation by LPS and associates with the presence of type 2 diabetes in obese humans. (**a**) Enriched or depleted (*p* < 0.01) KEGG-derived gene sets in ATMs of obese (HFD; *n* = 4 pools) vs lean (LFD; *n* = 4 pools) mice. (**b**, **d**) Regulation of these enriched or depleted gene sets in LPS-activated (*n* = 4) vs untreated BMDMs (*n* = 4) (**b**) or in CD14^+^ cells from obese diabetic (*n* = 6) vs obese non-diabetic individuals (*n* = 6) (**d**). Data presented in (**b**) are derived from GEO accession no. GSE53986 and in (**d**) from GSE54350. (**c**) Heat map showing expression of genes involved in inflammation in ATMs from obese vs lean mice presented as log_2_ ratio (SLR) difference with red representing upregulation and blue representing downregulation in ATMs of HFD- vs LFD-fed mice; *p < 0.05, **p < 0.01 and ***p < 0.001 for obese vs lean mice

To gain further insight into functional properties of metabolic and inflammatory rewiring found in ATMs, gene set enrichment analysis (GSEA) was performed using metabolic and inflammatory gene sets as input (ESM Table 3). Overall, far more gene sets were significantly (*p* < 0.01) enriched than depleted (30 vs 2) in ATMs from obese vs lean mice and none of the depleted gene sets reflected metabolic pathways. Interestingly, metabolic gene sets including glycolysis and OXPHOS were strongly enriched in ATMs upon obesity (Fig. 2a). Noticeably, transcriptional regulation in ATMs in obesity was very distinct from classical macrophage activation by LPS. Although some pathways were similarly regulated, most metabolic routes, including OXPHOS, glycolysis and the pentose phosphate pathway, were less or inversely regulated in LPS-activated macrophages compared with ATMs from obese vs lean mice (Fig. 2b). In line with different metabolic regulation in ATMs vs classically activated macrophages, we found diverse regulation of various pro- and anti-inflammatory genes, including *Cd11c*, *Myd88*, *Arg1* and *Il-10* (also known as *Il10*) in ATMs from obese vs lean mice not resembling M1 (nor M2) macrophages (Fig. 2c). Interestingly, metabolic activation as found in the ATM part of obese adipose tissue was also apparent in CD14^+^ cells isolated from visceral adipose tissue of obese humans with diabetes compared with obese non-diabetic individuals (Fig. 2a, d).

### Functional consequences of metabolic activation of ATMs in obesity

**Fig. 3:**
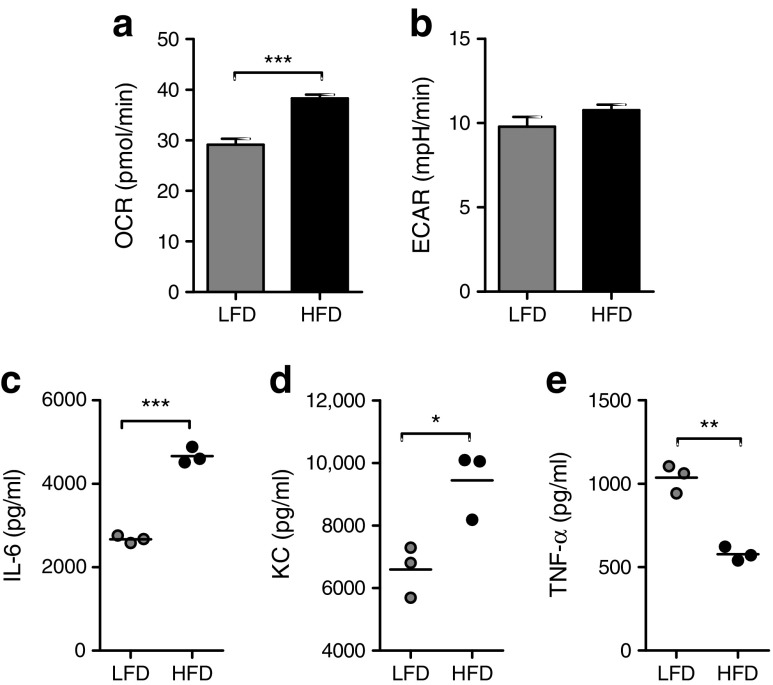
Functional consequences of metabolic activation in ATMs during obesity. (**a**, **b**) Basal OCR (**a**) or ECAR (**b**) of freshly isolated ATMs from obese (HFD) or lean (LFD) mice. (**c**–**e**) IL-6 (**c**), KC (**d**) or TNF-α (**e**) secretion over 24 h by ATMs from HFD- or LFD-fed mice. The ATMs (*n* ≥ 3) were plated from a pool of nine (LFD) or five (HFD) mice. Data are presented as means (**c**, **d**, **e**) ± SEM (**a**, **b**). *p < 0.05, **p < 0.01 and ***p < 0.001

Next, we evaluated whether transcriptional changes in ATMs translated into differences in energy metabolism. To that end, OXPHOS and glycolysis rates of freshly isolated ATMs were examined by measuring OCR and ECAR. In line with increased expression of metabolic genes, ATMs from obese mice displayed higher rates of OXPHOS (Fig. 3a). Moreover, a higher glycolytic rate was seen in ATMs from obese mice (Fig. 3b). Although this difference did not reach statistical significance, it corroborated the significantly higher levels of lactate in supernatant fractions of ATMs from obese vs lean mice after a 24 h culture period (Fig. 1h). As expected based on previous findings [2, 19–21], ATMs from obese mice produced more IL-6 and KC than macrophages isolated from lean adipose tissue (Fig. 3c, d). In contrast, less TNF-α was secreted by ATMs from obese vs lean mice (Fig. 3e), even though expression of *Tnf* was increased in ATMs (Fig. 2c). Of note, neither IL-1β nor IL-10 could be detected in the supernatant fractions of ATMs.

### Exposing macrophages to adipose tissue explants ex vivo dose-dependently induces metabolic activation and cytokine release

**Fig. 4:**
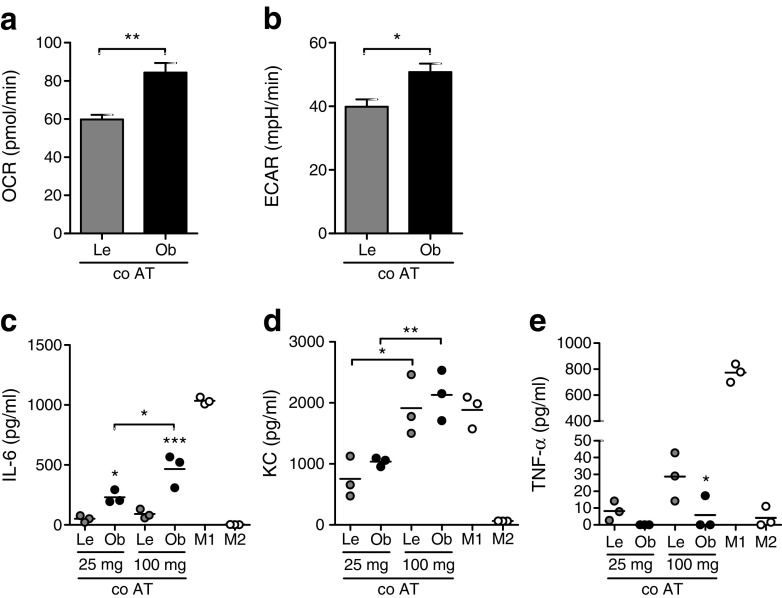
Dose-dependent adipose tissue-induced activation of macrophages ex vivo. (**a**, **b**) Basal OCR (**a**) and ECAR (**b**) of BMDMs co-cultured (co AT) with 100 mg of obese (Ob) or lean (Le) adipose tissue for 3 days. (**c**–**e**) Secretion of IL-6 (**c**), KC (**d**) or TNF-α (**e**) by BMDMs exposed to lean or obese adipose tissue (AT), or activated by LPS (M1) or IL-4 (M2). Effects of diet (obese vs lean) and dose of adipose tissue (25 mg vs 100 mg) were tested for significance. *n* ≥ 3 for all experiments. Data are presented as means (**c**, **d**, **e**) ± SEM (**a**, **b**); *p < 0.05, **p < 0.01

To examine whether changes in the adipose tissue environment drive metabolic and inflammatory activation of ATMs in obesity, we co-cultured BMDMs with lean or obese adipose tissue for 3 days, removed the adipose tissue and examined the adipose tissue-induced activation of BMDMs in comparison with classically (LPS) or alternatively (IL-4) activated BMDMs both directly (metabolism) and after 24 h in fresh medium (cytokines). As observed with the ATMs from obese mice, BMDMs exposed to obese adipose tissue exhibited enhanced glycolysis and OXPHOS compared with BMDMs exposed to lean adipose tissue, reflected by increased OCR (Fig. 4a) and ECAR (Fig. 4b). Moreover, BMDMs exposed to obese adipose tissue secreted more IL-6 and KC and less TNF-α than BMDMs exposed to lean adipose tissue (Fig. 4c–e), like ATMs from obese vs lean mice (Fig. 3c–e). Interestingly, inflammatory activation was found to be dose-dependently induced by the adipose tissue explants and appeared distinct from either LPS- or IL-4-activated macrophages (Fig. 4c–e). It is worth noting that metabolic rewiring followed a similar trend, with a dose-dependent increase in glycolysis in macrophages exposed to either lean or obese adipose tissue (ESM Fig. 1a) and higher OXPHOS specifically in macrophages exposed to 100 mg of obese adipose tissue (ESM Fig. 1b). Our observation of dose-dependent adipose tissue-induced activation of macrophages, different from classical activation, points to a role for specific environmental cues in driving the unique ATM signatures. Adipose tissue is a source of leptin [22] and lactate [23] which are known to affect immune cell metabolism and function [24–26]. Despite higher levels of both lactate and leptin in the supernatant fractions of adipose tissue explants isolated from obese vs lean mice (ESM Fig. 1c,d), application of neither leptin nor lactate could induce metabolic activation comparable with that seen in macrophages exposed to obese adipose tissue (ESM Fig. 1e,f). Of note, as with ATMs, neither IL-1β nor IL-10 were detected in supernatant fractions of BMDMs after exposure to adipose tissue.

### Glycolysis largely controls cytokine release by ATMs in obesity

**Fig. 5:**
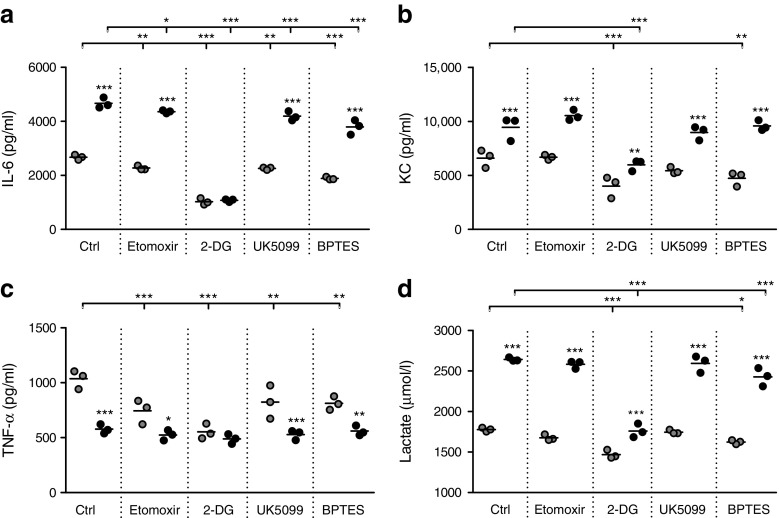
Glycolysis largely controls cytokine release by ATMs during obesity. Secretion of IL-6 (**a**), KC (**b**), TNF-α (**c**) or lactate (**d**) by ATMs isolated from lean (grey circles) or obese (black circles) mice. Cytokines were measured basally (Ctrl) or upon stimulation of ATMs with various metabolic inhibitors for 24 h. Effects of diet (obese vs lean) and treatment (metabolic inhibitors vs control) were tested for significance. Secretion of TNF-α by ATMs from obese mice tended to be reduced upon treatment with 2-DG (*p* = 0.06). ATMs (*n* = 3) were plated from a pool of 9 LFD-fed mice and 5 HFD-fed mice. Data are presented as means; *p < 0.05, **p < 0.01 and ***p < 0.001 for indicated comparisons (vs Ctrl obese or lean as shown) or for obese vs lean within each treatment

To examine the contribution of metabolic routes for cytokine production by ATMs we measured IL-6, KC and TNF-α upon inhibition of glucose uptake (2-DG), fatty acid oxidation (Etomoxir), glucose oxidation (UK5099) or glutamine influx into the tricarboxylic acid cycle (BPTES). Minimal morphological changes were observed in ATMs after treatment with these compounds. Although several metabolic routes appeared to contribute to cytokine release in ATMs from lean mice, overall, 2-DG treatment had the most profound effects on cytokine release in ATMs from both lean and obese mice (Fig. 5a–c). Importantly, inhibition of glycolysis with 2-DG abolished basal differences in cytokine release by ATMs from obese vs lean mice, demonstrating that glycolysis makes an important contribution to the increased inflammatory cytokine production by ATMs in obese adipose tissue (Fig. 5a–c). By competing with glucose, 2-DG may reduce both lactate production and glucose oxidation. Because UK5099 affected cytokine release by ATMs to a lesser extent than 2-DG, likely anaerobic glycolysis and not glucose oxidation is important for cytokine secretion by ATMs, especially in the obese state. Indeed 2-DG was the only inhibitor that strongly reduced lactate secretion by ATMs (Fig. 5d), supporting a link between lactate production and cytokine release by ATMs, particularly in obese conditions.

### Myeloid-specific Hif-1α does not affect adipose tissue inflammation in HFD-fed mice

**Fig. 6:**
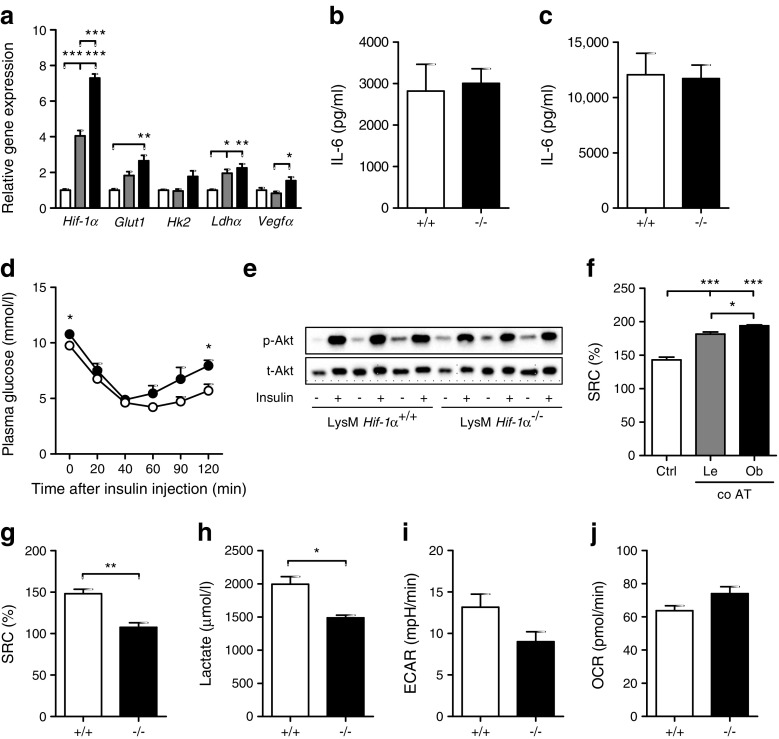
Myeloid-specific absence of HIF-1α does not affect adipose tissue inflammation in HFD-fed mice. (**a**) Fold change expression of *Hif-1α* and its target genes in BMDMs held in L929-conditioned medium (white bars) or exposed to 100 mg lean adipose tissue explant (grey bars) or obese adipose tissue explant (black bars) for 3 days. Starting quantities were used for normalisation against *36b4*. (**b**, **c**) IL-6 production by epidydimal adipose tissue isolated from LysM *Hif-1α*^+/+^ (+/+) or LysM *Hif-1α*^−/−^ (−/−) mice, either unstimulated (**b**) or stimulated with 10 ng/ml LPS for 24 h (**c**). (**d**) Glucose measured in plasma of LysM *Hif-1α*^+/+^ (white circles) or LysM *Hif-1α*^−/−^ (black circles) mice upon the injection of insulin at 0 min. (**e**) Total Akt (t-Akt) and p-Akt protein levels in epidydimal adipose tissue from LysM *Hif-1α*^+/+^ or LysM *Hif-1α*^−/−^ mice unstimulated (−) or stimulated with insulin (+) for 20 min. (**f**) SRC as percentage increase from basal OCR in BMDMs in 5% (vol./vol.) L929 (Ctrl) or upon 3 days of co-culture (co AT) with 100 mg adipose tissue isolated from lean (Le) or obese (Ob) mice. (**g**) SRC as percentage increase from basal OCR in BMDMs isolated from LysM *Hif-1α*^+/+^ (+/+) or LysM *Hif-1α*^−/−^ (−/−) mice. (**h**) Lactate secretion over 24 h by BMDMs isolated from LysM *Hif-1α*^+/+^ (+/+) or LysM *Hif-1α*^−/−^ (−/−) mice. (**i**, **j**) Basal ECAR (**i**) and OCR (**j**) in BMDMs from LysM *Hif-1α*^+/+^ (+/+) or LysM *Hif-1α*^−/−^ (−/−) mice. Basal ECAR were lower in BMDMs of LysM *Hif-1α*^−/−^ vs LysM *Hif-1α*^+/+^ mice (although the difference did not reach statistical significance, *p* < 0.051). For the in vivo study, *n* = 8 animals per genotype/diet were included. *n* = 3 for all in vitro experiments. Data are presented as means ± SEM. *p < 0.05, **p < 0.01 and ***p < 0.001

Key metabolic regulators such as AMPK and HIF-1α link intracellular metabolism to inflammatory activation [14, 27]. Metabolic stress-reactive AMPK enhances OXPHOS and fatty acid oxidation upon its activation through phosphorylation and was clearly induced in macrophages in an obese adipose tissue environment (ESM Fig. 2a). The transcription factor HIF-1α, a regulator of genes involved in glycolysis, was predicted to be activated in ATMs part of obese adipose tissue as well (Ingenuity Pathway Analysis: *z* score 2.460, *p* value overlap <0.01). Moreover, *Hif-1α* itself and its target genes were upregulated in macrophages in an obese adipose tissue environment, with higher levels in BMDMs exposed to obese vs lean adipose tissue (Fig. 6a). Unexpectedly, however, deletion of *Hif-1α* in myeloid cells did not affect the inflammatory state of obese adipose tissue after 8 weeks of HFD feeding, as demonstrated by equal IL-6 secretion by complete adipose tissue isolated from LysM *Hif-1α*^*−/−*^ and LysM Hif-1α^+/+^ mice, both basally (Fig. 6b) and upon LPS stimulation (Fig. 6c). Analysis of macrophage and inflammatory marker expression (Cd68, Cd11c, Cd206) in the adipose tissue revealed no protection against adipose tissue inflammation in HFD-fed mice lacking Hif-1α in the myeloid compartment (data not shown). Moreover, no robust differences were found in either total body insulin sensitivity (Fig. 6d) or in adipose tissue-specific insulin sensitivity (Fig. 6e) in LysM *Hif-1α*^−/−^ vs LysM *Hif-1α*^+/+^ mice after 8 weeks of HFD feeding. We did observe slightly higher glucose levels, as well as a steeper rebound phase of the insulin tolerance test, in LysM *Hif-1α*^−/−^ vs LysM *Hif-1α*^+/+^ mice (Fig. 6d), pointing to impaired fasting glucose. However, the LysM *Hif-1α*^−/−^ mice were heavier than the LysM *Hif-1α*^+/+^ mice (ESM Fig. 2b,c), resulting in higher adipose tissue weight (ESM Fig. 2d), which may have accounted for the higher fasting glucose. It is worth noting that differences in body weight could not be explained by food intake, which was similar in both genotypes (data not shown), and may rather be due to differences in locomotor activity or energy absorption, although this requires further study.

We observed increased spare respiratory capacity (SRC) of macrophages in an adipose tissue environment compared with control macrophages (Fig. 6f), indicative of enhanced cellular flexibility and capacity to manage stressful situations [28, 29]. Interestingly, macrophages lacking Hif-1α had lower SRC than wild-type BMDMs (Fig. 6g) and appeared to be less glycolytic (Fig. 6h, i). The absence of compensation for lower glycolytic rates by *Hif-1α*^−/−^ BMDMs (i.e. by increasing oxygen consumption) (Fig. 6j), is suggestive of reduced metabolic capacity and flexibility in the absence of Hif-1α and this may outweigh the potential anti-inflammatory effects of lower glycolysis in an adipose tissue environment.

## Discussion

In obesity, macrophages fuel adipose tissue inflammation, promoting the development of insulin resistance and type 2 diabetes [30]. The inflammatory state of ATMs has been studied extensively. To our knowledge, however, we are the first to measure real-time metabolic fluxes in freshly isolated ATMs. Interestingly, our data strongly point to unique metabolic activation that drives cytokine release by ATMs in obesity, resembling neither the metabolic nor inflammatory signatures seen in M1- or M2-primed macrophages or peritoneal macrophages.

Cumulative evidence from the field of immunology shows that robust metabolic rewiring fuels differential inflammatory activation of macrophages. On the one hand, M2 macrophages require OXPHOS for responses, whereas M1 macrophages rely on aerobic glycolysis [11, 12]. In contrast to these two extremes, we found that macrophages in an adipose tissue environment adopt a unique metabolic profile in obesity, characterised by activation of various metabolic routes including both OXPHOS and glycolysis. Metabolic and inflammatory adaptations in obesity were specific for ATMs, as no metabolic rewiring was found in peritoneal macrophages. In line with our finding of unique metabolic rewiring in ATMs and supportive of various studies reporting diverse inflammatory activation of ATMs in obese adipose tissue [9, 16, 31, 32], we found inflammatory activation of ATMs to be different from that of classically activated macrophages.

Interestingly, macrophages co-cultured with obese adipose tissue developed similar phenotypical adaptations in a dose-dependent manner, suggestive of obesity-induced changes in the adipose tissue microenvironment shaping the ATM phenotype. Indeed, the composition of adipose tissue is importantly affected in obesity, with resultant adipocyte hypertrophy and both accumulation and phenotypical changes of immune cells including macrophages. In our co-culture system we have used lean and obese adipose tissue explants of equal weight. This may not have accounted for all the shifts in relative cell numbers occurring in obese adipose tissue, yet strongly points toward the existence of divergent factors secreted by obese vs lean adipose tissue that may critically influence the macrophage phenotype in a dose-dependent manner. Potential factors may include adipokines, cytokines, fatty acids or other metabolites [30]. Interestingly, leptin [24, 25] and lactate [26] have been shown capable of remodelling intracellular metabolism and changing the inflammatory state of macrophages. We found both to be secreted more by obese adipose tissue than by lean adipose tissue, yet neither leptin nor lactate induced metabolic rewiring similar to that seen in macrophages in an adipose tissue environment. Additionally, differences in cell death between lean and obese adipose tissue in vivo may have an effect on metabolic rewiring in macrophages. Most likely, a mixture of signals is responsible for shaping ATM metabolic phenotypes in the lean and obese state, although this needs further investigation.

Importantly, metabolic activation of ATMs contributes to their inflammatory cytokine release. First, metabolically active ATMs from obese mice secreted far more IL-6 and KC than the less metabolically active ATMs isolated from lean mice. Second, we found that interference with metabolic routes directly affected cytokine release by ATMs. Especially in ATMs from lean mice, several metabolic routes including fatty acid oxidation, glycolysis and glutaminolysis contribute to cytokine release. Glycolysis appears to play a dominant role in fuelling the inflammatory trait of ATMs from obese adipose tissue, since inhibiting glycolysis with 2-DG almost completely abolished the greater basal cytokine secretion by ATMs from obese vs lean mice.

Our finding of lower TNF-α secretion by ATMs from obese vs lean mice was unexpected, as was the lower level of TNF-α in supernatant fractions of macrophages exposed to obese vs lean adipose tissue. Despite lower cytokine levels, we found *Tnf* upregulated at the mRNA level. A similar discrepancy between mRNA and protein level has been reported in ATMs before [10]. In obese adipose tissue, however, macrophage influx and proliferation as well as an increase in other immune cell populations likely overrules lower TNF-α secretion per macrophage and might be responsible for higher TNF-α levels found in the adipose tissue and circulation of obese individuals [6, 33–35]. Alternatively, enhanced autocrine TNF signalling in ATMs may explain the lower levels of TNF-α measured in ATM supernatant fractions.

Next to cytokine release, intracellular metabolism most likely controls several other macrophage functions. For example, OXPHOS has been found to contribute to phagocytosis by human monocytes [36], and lysosomal biogenesis and function in T cells [37]. Both phagocytic and lysosomal genes were found to be strongly upregulated in ATMs of obese mice and in obese individuals with type 2 diabetes. Phagocytosis of dead adipocytes by macrophages [38–40] and lysosomal function of ATMs [10, 39] are considered to be important for maintaining adipose tissue homeostasis. Hence, the obesity-induced increase in OXPHOS might fuel ATM functions in expanding adipose tissue, not directly related to inflammatory cytokine release yet may greatly affect adipose tissue function.

Our data show that interfering with metabolic routes alters the inflammatory phenotype of ATMs and that glycolysis importantly contributes to inflammatory cytokine release by ATMs. Unexpectedly, however, myeloid-specific deletion of a previously identified key regulator of glycolysis, Hif-1α, did not alleviate inflammatory activation of ATMs during the early stages of obesity. Because mice lacking Hif-1α in myeloid cells were significantly heavier yet did not display increased adipose tissue inflammation or insulin resistance, one could speculate that HIF-1α may be partly protective for the development of obesity-induced adipose tissue inflammation, as has been reported before in mice fed an HFD for 18 weeks [41]. One might also hypothesise that during the earlier stages of HFD-induced obesity HIF-1α is important for controlling other metabolic properties of macrophages not related to cytokine production. For example, our data revealed a decreased SRC in BMDMs from *Hif-1α*^−/−^ mice, suggestive of a role for HIF-1α in maintaining metabolic flexibility of macrophages. Indeed, despite decreased capability of using glycolysis as an energy source, Hif-1α^−/−^ macrophages lack the flexibility to increase their oxidative capacity [15]. Metabolic flexibility is probably needed for ATMs, as we observed enhanced SRC in macrophages in an adipose tissue environment which was even further increased upon exposure to obese adipose tissue. Reduced metabolic flexibility in macrophages lacking Hif-1α in expanding adipose tissue might have overruled effects on cytokine release during the development of obesity.

In conclusion, we identified unique metabolic activation of ATMs in obesity, characterised by increased OXPHOS and glycolysis. Blocking metabolic routes in isolated ATMs led to the identification of glycolysis as a main contributor to their proinflammatory trait, especially in obesity. Interestingly, metabolic signatures, similar to those found in ATMs upon obesity, including the induction of OXPHOS and lysosomal genes, were observed in human macrophages isolated from adipose tissue of obese individuals with type 2 diabetes. Further understanding of metabolic programming in ATMs will most likely lead to novel therapeutic targets to modulate macrophage metabolism and curtail inflammatory responses that drive insulin resistance and type 2 diabetes in obese individuals.

## Electronic supplementary material

ESM (PDF 259 kb)

## Abbreviations

AMPK: AMP-activated protein kinase
ATM: Adipose tissue macrophage
BMDM: Bone marrow-derived macrophage
2-DG: 2-Deoxy glucose
ECAR: Extracellular acidification rate
GSEA: Gene set enrichment analysis
HFD: High-fat diet
HIF-1α: Hypoxia-inducible factor-1α
KC: Chemokine (C-X-C motif) ligand-1
LFD: Low-fat diet
LPS: Lipopolysaccharide
OCR: Oxygen consumption rate
OXPHOS: Oxidative phosphorylation
PCA: Principal component analysis
PS: Penicillin/streptomycin
qRT-PCR: Quantitative reverse-transcription PCR
SRC: Spare respiratory capacity
