# Supplementary material for: Unique metabolic activation of adipose tissue macrophages in obesity promotes inflammatory responses
Source: Diabetologia. 2018 Jan 14;61(4):942–53. doi: 10.1007/s00125-017-4526-6 (PMC6448980; doi:10.1007/s00125-017-4526-6)
Supplement: Supplementary file 1 — (PDF 259 kb) [file 125_2017_4526_MOESM1_ESM.pdf]

ESM Methods

Collection of adipose tissue and macrophages

After cervical dislocation, epididymal adipose tissue was removed and fixated for immunohistochemistry, fresh-frozen for RNA isolation, or processed sterile to separate the stromal vascular fraction (SVF) from adipocytes using collagenase digestion as described previously [1]. Adipose tissue macrophages (ATMs) were purified from the stromal vascular fraction by F4/80+ positive selection with magnetic beads (Milteny Biotec, Leiden, The Netherlands). Cell pellets were frozen directly for RNA isolation, or were re-suspended and brought into culture in RPMI 1640 (Lonza, Verviers, Belgium) supplemented with 10% vol./vol. FCS and 1% vol./vol. PS (RPMI/FCS/PS). Peritoneal macrophages were obtained by infusion and subsequent collection of ice-cold PBS from the abdominal cavity, and frozen directly or brought into culture in Dulbecco's modified eagle's medium (Lonza) supplemented with 10% vol./vol. FCS and 1% vol./vol. PS (DMEM/FCS/PS). Bone marrow cells were isolated from femurs and tibia of low-fat diet- or chow-fed mice following a standard protocol.

Extracellular flux analysis

Basal metabolic rates of ATMs or bone marrow-derived macrophages (BMDMs) seeded in quintuplicate were determined during four consecutive measurements in unbuffered Seahorse medium (8.3 g DMEM powder, 0.016 g phenol red and 1.85 g NaCl in 1 l milli-Q, pH set at 7.4 at 37 °C; sterile-filtered) containing 11 mmol/l glucose (ATMs) or 25 mmol/l glucose (BMDMs) and 2 mmol/l L-glutamine (ATMs & BMDMs). After basal measurements, three consecutive measurements were taken upon the addition of each 1.5 µmol/l oligomycin, 1.5 mmol/l FCCP, and 2 µmol/l antimycin together with 1 µmol/l rotenone. Pyruvate (1 mmol/l) was added together with FCCP to fuel maximal respiration. Spare Respiratory Capacity was calculated as percentage increase in OCR after FCCP injection, with basal OCR set at 100%. All compounds used during the Seahorse runs were acquired from Merck. Signals were normalized to relative DNA content in the wells using the Quant-iT™ dsDNA Assay Kit (ThermoFisher Scientific).

Western blot

Cells were lysed in RIPA buffer (ThermoFisher Scientific, Landsmeer, The Netherlands) enriched with protease and phosphatase inhibitors (Roche Diagnostics). Total protein was determined using a BCA protein assay (ThermoFischer Scientific) and for each sample 15 µg of protein was loaded on a 4-15% Mini-PROTEAN TGX Precast gel (Bio-Rad). Proteins were separated by SDS gel electrophoresis and transferred to a PVDF membrane (Bio-Rad) using the Transblot Turbo System (Bio-Rad). All incubations were in Tris-buffered saline with 0.1% vol./vol. Tween-20 (TBS-T), containing 5% weight/vol. dry milk (β-actin) or 5% weight/vol. bovine serum albumin (AMPK, phospho-AMPK, phospho-Akt). HRP-conjugated goat-anti-rabbit secondary Ab (Merck) was used 1:5000 in TBS-T with 5% weight/vol. dry milk. Blots were visualized using the ChemiDoc MP system (Bio-Rad) and Clarity ECL substrate (Bio-Rad).

RNA isolation and qPCR

Total RNA of ATMs and peritoneal macrophages was isolated with a RNeasy mini- or microkit (Qiagen, Venlo, The Netherlands). RNA of BMDMs was isolated using phenol/chloroform extraction. The RNA was reverse transcribed with the iScript cDNA synthesis kit (Bio-Rad). Real-time quantitative PCR was carried out with a SensiMix SYBR kit (Bioline, Alphen aan den Rijn, The Netherlands) in a CFX384 system (Bio-Rad). Relative expression levels were calculated after normalization against the housekeeping gene *36b4* using the $2^{-\Delta\Delta C_t}$ method (ATMs and peritoneal macrophages) or starting quantities (BMDMs). Primer sequences can be found in ESM Table 1. For qPCR analysis, four pools of ATMs from LFD-fed, and six pools of ATMs from HFD-fed mice were used. Peritoneal macrophages of seven individual mice on a LFD and six mice on a HFD were included.

Microarray analysis and interpretation

Purified total RNA (100 ng per sample) was labelled with the Whole-Transcript Sense Target Assay (Affymetrix, Santa Clara, CA, USA; P/N 900652) and hybridized to whole-genome Affymetrix Mouse Gene 1.1 ST arrays (Affymetrix). Quality control and data analysis pipeline have been described in detail previously [2]. Briefly, normalized expression estimates of probe sets were computed by the robust multiarray analysis (RMA) algorithm [3, 4] as implemented in the Bioconductor library AffyPLM. Probe sets were redefined according to Dai et al. [5] based on annotations provided by the Entrez Gene database, which resulted in the profiling of 21,187 unique genes (custom CDF v20). Differentially expressed probe sets (genes) were identified by using linear models (library limma) and an intensity-based moderated t-statistic [6, 7]. Probe sets that satisfied the criterion of $p<0.01$ were included for Ingenuity Pathway Analysis. The Affymetrix dataset (GSE53986) was reanalysed as described above. The Illumina BeadChip experiments (GSE56682 and GSE54350) were reanalysed by background correction followed by quantile normalization (neqc) [8], removal of unspecific probes [9], where after differentially expressed probes were identified using limma [6, 7]. Principle component analysis (PCA) was performed in R using the library mixOmics [10]. Changes in gene expression were related to biologically meaningful changes using gene set enrichment analysis [11]. Only gene sets comprising more than 15 and fewer than 500 genes were taken into account. For each comparison, genes were ranked on their t-value that was calculated by the moderated t-test. Statistical significance of GSEA results was determined using 1000 permutations. The library clusterProfiler was used for visualization and interpretation of the GSEA results [12].

GSE56682

Peritoneal macrophages: F4/80+ and CD11b+ selection
Alveolar macrophages: CD11b+ and CD11c+ selection
Splenic macrophages: F4/80+ and CD11b+ selection
Kupffer cells: F4/80+ and CD11b+ selection
Intestinal macrophages: F4/80+ CD11b+ selection

GSE54350

Adipose tissue macrophages: CD14+ selection
A total of *n*=6 subjects were used per group. The obese individuals part of each study group were age- and sex-matched.

GSE53986

Untreated (*n*=4) versus LPS-stimulated (*n*=4) (10 ng/mL for 24 h) bone marrow-derived macrophages.

ESM References

[1] Stienstra R, Dijk W, van Beek L, et al. (2014) Mannose-binding lectin is required for the effective clearance of apoptotic cells by adipose tissue macrophages during obesity. Diabetes 63: 4143-4153

[2] Lin K, Kools H, de Groot PJ, et al. (2011) MADMAX - Management and analysis database for multiple ~omics experiments. Journal of integrative bioinformatics 8: 160

[3] Irizarry RA, Hobbs B, Collin F, et al. (2003) Exploration, normalization, and summaries of high density oligonucleotide array probe level data. Biostatistics 4: 249-264

[4] Bolstad BM, Irizarry RA, Astrand M, Speed TP (2003) A comparison of normalization methods for high density oligonucleotide array data based on variance and bias. Bioinformatics 19: 185-193

[5] Dai M, Wang P, Boyd AD, et al. (2005) Evolving gene/transcript definitions significantly alter the interpretation of GeneChip data. Nucleic acids research 33: e175

[6] Ritchie ME, Phipson B, Wu D, et al. (2015) limma powers differential expression analyses for RNA-sequencing and microarray studies. Nucleic acids research 43: e47

[7] Sartor MA, Tomlinson CR, Wesselkamper SC, Sivaganesan S, Leikauf GD, Medvedovic M (2006) Intensity-based hierarchical Bayes method improves testing for differentially expressed genes in microarray experiments. BMC bioinformatics 7: 538

[8] Shi W, Oshlack A, Smyth GK (2010) Optimizing the noise versus bias trade-off for Illumina whole genome expression BeadChips. Nucleic acids research 38: e204

[9] Barbosa-Morais NL, Dunning MJ, Samarajiwa SA, et al. (2010) A re-annotation pipeline for Illumina BeadArrays: improving the interpretation of gene expression data. Nucleic acids research 38: e17

[10] Le Cao KA, Gonzalez I, Dejean S (2009) integrOmics: an R package to unravel relationships between two omics datasets. Bioinformatics 25: 2855-2856

[11] Subramanian A, Tamayo P, Mootha VK, et al. (2005) Gene set enrichment analysis: a knowledge-based approach for interpreting genome-wide expression profiles. Proceedings of the National Academy of Sciences of the United States of America 102: 15545-15550

[12] Yu G, Wang LG, Han Y, He QY (2012) clusterProfiler: an R package for comparing biological themes among gene clusters. Omics : a journal of integrative biology 16: 284-287

ESM tables and figures

ESM Table 1

Primer sequences used to measure gene expression levels with qPCR.

Gene	3' primer	5' primer
Cd11c	CTGGATAGCCTTTCTTCTGCTG	GCACACTGTGTCCGAACTCA
Cd206	GCTTCCGTCACCCTGTATG	GTGTGTCATTCTTACACTCCCAT
Cd36	TCCAGCCAATGCCTTTGC	TGGAGATTACTTTTCAGTGCAGAA
Cd68	CCAATTCAGGGTGGAAGAAA	CTCGGGCTCTGATGTAGGTC
Glut1	GGTCACCATCTTGGAGCTGTTC	ACCTGCCTTCTCGAAGATGCT
Hk2	CGTGTCCCTACCTTTGGGTT	CCAGGTCAAACTCCTCTCGC
Hif-1α	ACCTTCATCGGAAACTCCAAAG	CTGTTAGGCTGGGAAAAGTTAGG
Ldhα	CATTGTCAAGTACAGTCCACACT	TTCCAATTACTCGGTTTTTGGGA
Lipa	TGTTCGTTTTCACCATTGGGA	CGCATGATTATCTCGGTCACA
Pdk4	TCTACAAACTCTGACAGGGCTTT	CCGCTTAGTGAACACTCCTTC
Plin2	GCCTCTCAACTGGCTGGTAG	ACAGCAAAAGGGGTCATCTG
Vegfα	CGGGCCTCGGTTCCAG	CTGGGACCACTTGGCATGG
36B4	ATGGGTACAAGCGCGTCCTG	GCCTTGACCTTTTCAGTAAG

ESM Table 2

KEGG-derived biosystems used for principle component analyses.

Gene set	BSID
Inflammatory activation	511699 & 511700
OXPHOS	83142
Glycolysis	198376
Amino acid metabolism	101155 & 83157

ESM Table 3

KEGG networks used as input for gene set enrichment analyses.

KEGG pathway	Network
Metabolism	1.0-1.8
Genetic information processing	2.3-2.4
Environmental information processing	3.1-3.2
Cellular processes	4.1
Organismal systems	5.1-5.2
Human diseases	6.7

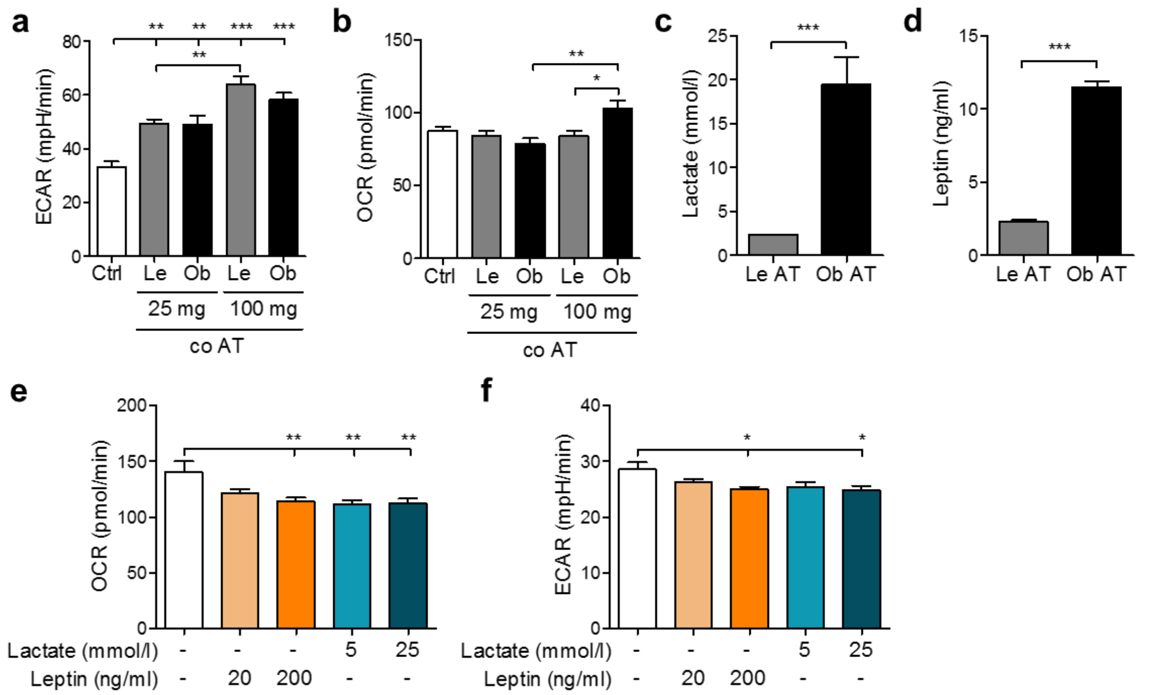

ESM Fig. 1 Leptin and lactate are secreted by obese adipose tissue yet do not skew macrophage metabolism towards the adipose tissue-induced phenotype

(**a**,**b**) Basal extracellular acidification rate (ECAR) (**a**) or oxygen consumption rate (OCR) (**b**) measurements of BMDMs in 5% vol./vol. L929 (Ctrl) or exposed to 25 mg or 100 mg lean (Le) or obese (Ob) adipose tissue (AT) for 3 days. (**c**,**d**) Leptin (**c**) and lactate (**d**) measured in supernatants of a 3-day culture of 100 mg lean or obese adipose tissue. (**e**,**f**) OCR (**e**) and ECAR (**f**) of BMDMs exposed to leptin or lactate for 24 h were compared to BMDMs exposed to 5% vol./vol. L929 (-). n=3 for all experiments. Data are presented as means ± SEM. * p<0.05, ** p<0.01, *** p<0.01.

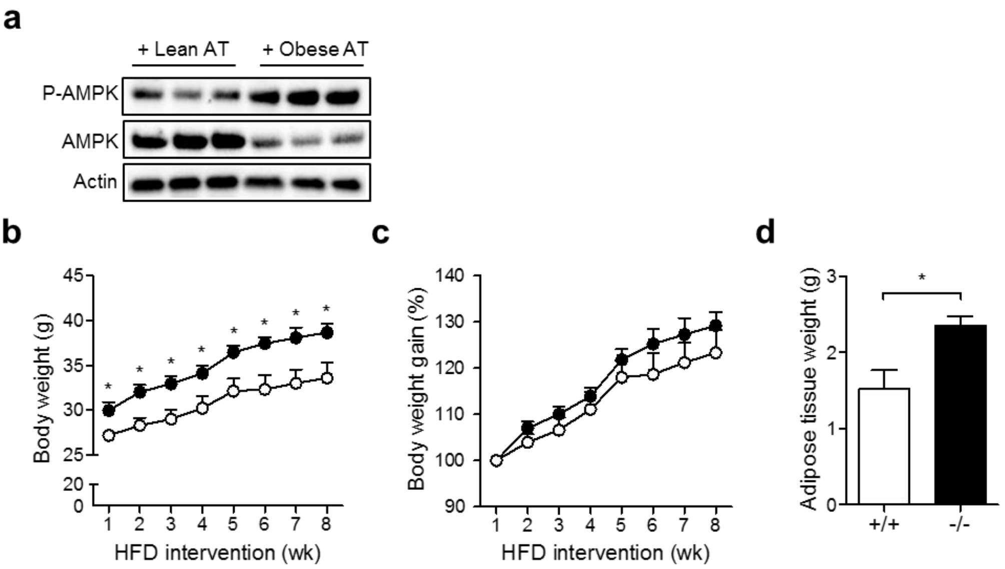

ESM Fig. 2 Higher bodyweight and adipose tissue weight in LysM *Hif-1α*$^{-/-}$ vs LysM *Hif-1α*$^{+/+}$ mice fed a HFD for 8 weeks

(**a**) Presence of phospho-AMPK (P-AMPK) and AMPK protein levels in BMDMs exposed to 100 mg of obese or lean adipose tissue (AT) for 3 days. Actin serves as loading control. (**b,c**) Bodyweight (**b**) and bodyweight gain (**c**) of LysM *Hif-1α*$^{+/+}$ and LysM *Hif-1α*$^{-/-}$ mice over the 8-week high-fat diet (HFD) intervention period. (**d**) Epidydimal adipose tissue weight of LysM *Hif-1α*$^{+/+}$ and LysM *Hif-1α*$^{-/-}$ mice after 8 weeks on a HFD. White bars or circles; LysM *Hif-1α*$^{+/+}$, black bars or circles; LysM *Hif-1α*$^{-/-}$. Data are presented as means ± SEM. * p<0.05.
